# Dentoskeletal changes and anteroposterior improvements in skeletal class III malocclusion treated with MEAW: A retrospective study

**DOI:** 10.1371/journal.pone.0340197

**Published:** 2026-01-02

**Authors:** Phi Ngoc Quang Tran, Anh Hoang Cu, Minh Ngoc Thuy Tran, Vy Ngoc Thuy Tran

**Affiliations:** 1 Department of Orthodontics and Pedodontics, Faculty of Dentistry, Van Lang University, Ho Chi Minh City, Vietnam; 2 Department of Orthodontics, Faculty of Dentistry, Pham Ngoc Thach University, Ho Chi Minh City, Vietnam; 3 Department of Periodontology and Implantology, Faculty of Dentistry, Van Lang University, Ho Chi Minh City, Vietnam; Universidade Federal do Rio de Janeiro, BRAZIL

## Abstract

**Background:**

Skeletal Class III malocclusion is a severe dentofacial deformity that often requires surgical correction, but the associated risks have increased interest in non-surgical alternatives. The multiloop edgewise archwire (MEAW) technique has been used as a conservative option. The aim of this study was to investigate the dentoskeletal changes associated with the MEAW technique in treating skeletal Class III malocclusion, with a particular focus on improvements in anteroposterior bimaxillary relationships and comparison with surgical outcomes.

**Methods:**

This retrospective study included 60 patients with skeletal Class III malocclusion, comprising 30 treated with the MEAW technique and 30 matched patients who underwent orthognathic surgery. Dentoskeletal parameters were assessed on pre- and post-treatment lateral cephalograms. Pearson’s correlation analysis and multiple linear regression were performed to identify factors associated with improvements in anteroposterior relationships.

**Results:**

Significant dental and skeletal changes were observed, including alterations in incisor inclination and bimaxillary measurements (*p* < 0.05). Post-treatment, anteroposterior relationships improved significantly, with changes strongly associated with increases in the BaNA angle and decreases in the SNB angle. However, compared to the surgical group, the MEAW group demonstrated less pronounced improvements in anteroposterior bimaxillary relationships. Regression analysis identified the BaNA angle and incisor inclination as key contributors.

**Conclusions:**

Although the effect is less pronounced than in the case of surgical intervention, the MEAW technique effectively improves anteroposterior bimaxillary relationships in skeletal Class III malocclusion. These findings suggest that MEAW is a valuable non-surgical alternative for selected skeletal Class III patients.

## Introduction

Class III malocclusion is typically defined as an abnormal dental arch relationship, which may or may not be associated with anteroposterior and vertical skeletal discrepancies [1]. Its prevalence varies widely across racial groups, ranging from 0% to 27.6%, with higher rates observed in Asian populations [2].

Adult skeletal Class III malocclusion represents one of the most severe and challenging conditions to treat, often requiring surgical intervention due to the complex interplay of cranial base abnormalities, skeletal structures, and dental components of the maxilla and mandible [3]. However, surgical treatment has limitations, including high costs, potential complications, and adverse effects such as neurosensory deficits and loss of tooth sensitivity [4,5]. These challenges frequently drive patients to seek non-surgical alternatives, despite their limitations in fully correcting the underlying skeletal discrepancies.

The multiloop edgewise archwire (MEAW) technique was originally developed by Kim for open bite correction and later adapted by Sato for treating Class III malocclusion [6,7]. In addition to dentoalveolar compensation as a camouflage effect, this method modifies bimaxillary relationships by repositioning the mandible posteriorly through occlusal plane adjustments. According to craniofacial dynamic theory, the functional forces generated during mastication are transmitted to the cranial base via the masticatory muscles and temporomandibular joint, influencing cranial bone flexion and extension movements. These biomechanical interactions, in turn, affect the orientation of the occlusal plane and contribute to the development of skeletal discrepancies, including Class III malocclusion [7–9]. Conversely, therapeutic alteration of the occlusal plane, as achieved with MEAW, may modulate these force vectors, promoting a more favorable mandibular position [7–9]. However, evidence supporting mandibular repositioning and skeletal modifications in the correction of skeletal Class III malocclusion using the MEAW technique remains inconclusive, and their impact on final treatment outcomes is still uncertain [10–12].

This study aimed to investigate the dentoskeletal changes associated with the MEAW technique in the treatment of skeletal Class III malocclusion, with a particular focus on whether it contributes to improvements in anteroposterior bimaxillary relationships and its potential effectiveness in modifying skeletal discrepancies.

## Materials and methods

### Patient selection

Sample size calculation was performed using G*Power 3.1 [13]. The calculation was based on published data [14] reporting ANB angle changes in skeletal Class III malocclusion patients treated with camouflage therapy (mean change = 0.92°, SD = 1.9°), at least 28 subjects were required for the MEAW group to achieve 80% statistical power at a significance level of 0.05. To further support this estimation, a calculation using Wits appraisal changes (mean change = 2.28 mm, SD = 2.9 mm) from the same dataset [14] yielded a required minimum of 15 participants. To ensure comparability between treatment modalities, an equal number of surgical patients were recruited.

Medical records were accessed on 15th January 2024 solely for research purposes. The authors had no access to information that could identify individual participants. Patients with skeletal Class III malocclusion who were treated at the Department of Orthodontics and Pedodontics, Faculty of Dentistry, Van Lang University, between July 2021 and October 2023 were retrospectively identified and assigned into two groups based on their treatment modality: the MEAW group and the surgical group. The MEAW group was consecutively selected according to the inclusion criteria. The surgical group was selected using the same criteria and matched to the MEAW group according to malocclusion type, presence of anterior open bite, dentoalveolar characteristics such as severe negative overjet, bilateral or unilateral posterior crossbite, and severe dental crowding. The inclusion criteria for both groups were as follows:

1. Dental Class III malocclusion;
2. Skeletal Class III relationship (ANB < 0 ^0^ and Wits appraisal ≤ –2 mm);
3. Males >18 years, females >16 years;
4. Skeletal maturity confirmed by cervical vertebral stage CS6;
5. Absence of syndromic or medically compromised conditions;
6. No history of prior surgical intervention;
7. Presence or absence of mandibular deviation;
8. No mandibular functional shift;
9. Completion of orthodontic treatment;
10. Availability of complete records (cephalograms, photographs, and cast models) before and after treatment.

The MEAW group consisted of 30 patients (4 males, 26 females; mean age: 24.4 ± 6.4 years) with skeletal Class III malocclusion treated using the MEAW technique. Most patients in the MEAW group were treated without extractions; however, two patients required the extraction of the lower first premolars and upper second premolars. The surgical group consisted of 30 patients (16 males, 14 females; mean age: 22.7 ± 4.2 years) who received fixed appliance orthodontic treatment combined with orthognathic surgery. Surgical interventions consisted of either bimaxillary surgery (12 patients) or mandibular setback surgery alone (18 patients). Extractions were performed as needed: 8 patients had upper first premolars extracted, 4 had both upper first and lower second premolars extracted, and the remaining patients were treated without extractions.

Baseline equivalence between the MEAW and surgical groups was assessed by comparing pre-treatment variables, including age, bimaxillary skeletal parameters, and dental measurements (S1 Table). No significant differences were found between the groups for most variables (*p* > 0.05), except for mandibular length, which was significantly greater in the surgical group (*p* < 0.01). Accordingly, mandibular length, sex, and tooth extraction status were treated as covariates in the adjusted intergroup comparisons.

### Ethics and consent

The retrospective study was approved by the Ethics Committee of Van Lang University on November 3, 2023 (approval number 23/2023/HĐĐĐ-IRB-VN01.078) in accordance with the ethical principles outlined in the 1964 Declaration of Helsinki. Written informed consent was obtained from all participants prior to enrollment. The study was conducted and reported in accordance with the STROBE (Strengthening the Reporting of Observational Studies in Epidemiology) guidelines (S1 File).

### Treatment procedures

Surgical intervention was generally recommended for patients with marked skeletal discrepancies (e.g., Wits < –11 mm without transverse/vertical problems, or Wits < –5 mm when combined with other skeletal deformities) [15]. The MEAW technique was mainly indicated for borderline or moderate Class III cases [16]. Final treatment choice was determined by patients after counseling regarding risks, benefits, and limitations. As a result, a few patients with severe discrepancies chose MEAW instead of surgery. All orthodontic treatments were performed by a single experienced clinician with more than 10 years of practice. Surgical interventions were performed by a maxillofacial surgical team within the same institution and study period. The average treatment duration for the MEAW group was approximately 28 months, while the surgical group generally had a shorter overall treatment period.

#### a) Protocols of MEAW treatment.

The MEAW treatment in this study was based on the standard guidelines established by Sadao Sato [7]. Fixed appliances were bonded using RICKETTS Classic brackets (Rocky Mountain Orthodontics (RMO), USA) with a 0.018 × 0.025-inch slot. Compatible MEAW/GEAW archwires, including Elgiloy 0.016” × 0.022” and Gum Metal 0.017” × 0.022” (RMO, USA) were used.

The MEAW protocol is summarized in S1 Fig and S2 Fig. In brief, it involved five main steps: (1) initial alignment to enable archwire insertion; (2) elimination of occlusal interferences through molar tip-back activation; (3) mandibular repositioning using step-up bends combined with intermaxillary elastics; (4) reconstruction of the mandibular plane through controlled tooth movements; and (5) finishing to achieve a stable functional occlusion. Treatment procedures were adjusted according to the vertical skeletal pattern, classified as low- or high-angle based on the lower facial height (LFH) index [16,17]. Those within the normative range were managed using the high-angle protocol. In Vietnam, the normative LFH index is defined as 42°–51° (Table 1).

**Table 1:** Cephlometric measurements, definitions and Vietnamese norm values (mean ± SD).

Measurements		Definition	Norm (Mean ± SD)
Cranial base measurements			
1	Anterior cranial length, CC-Na (mm)	Distance from CC (crossing point of facial axis and Ba-N plane) to Nasion	53.4 ± 3.0
2	Posterior cranial length, Cp–PtV (mm)	Distance from Cp (most posterior point on the head of condylar head) to PtV	32.5 ± 3.6
3	Cranial deflexion, BaN–FH (^0^)	Angle between BaN and FH planes	31.1 ± 2.3
Maxillary measurements			
4	Maxillary protrusion angle, BaNA (^0^)	Angle between Ba–N and N–A planes	60.4 ± 5.2
5	SNA (^0^)	Angle between S–N and N–A planes	83.2 ± 4.2
6	Effective maxillary length, CoA (mm)	Distance from Co (most superior point on the head of condylar head) to A point	89.8 ± 5.2
Mandibular measurements			
7	SNB (^0^)	Angle between S–N and N–B planes	80.8 ± 4.3
8	Mandibular length, XiPm (mm)	Distance from Xi point (centroid reference for the mandibular ramus) to Pm	66.8 ± 4.1
9	Facial axis angle, PtGn–BaN (^0^)	Angle between Pt–Gn and Ba–N planes	95.4 ± 3.9
10	Modified Y axis, SN–SGn (^0^)	Angle between S–N and S–Gn planes	66 ± 4
Anteroposterior relationships			
11	Convexity, A–NPog (mm)	Distance from A to N–Pog plane	1.5 ± 1.5
12	Wits appraisal, Wits (mm)	Distance between points A and B projected onto the occlusal plane	0.8 ± 2.5
13	ANB (^0^)	Angle between N–A and N–B planes	2 ± 2
Vertical relationships			
14	Lower facial height, LFH (^0^)	Angle between ANS–Xi and Xi–Pm planes	46.5 ± 4.5
15	Frankfort-mandibular plane angle, FMA (^0^)	Angle between FH and mandibular planes	23.8 ± 5.2
Teeth and occlusal plane			
16	Upper incisor inclination, U1–PP (^0^)	Angle between the upper incisor axis and palatal plane (ANS–PNS)	113.8 ± 8
17	Incisor mandibular plane angle, IMPA (^0^)	Angle between the lower incisor axis and mandibular plane	87.4 ± 5.9
18	Interincisal angle, U1–L1 (^0^)	Angle between the upper incisal axis and the lower incisal axis	123.8 ± 8.8
19	Overbite (mm)	Vertical overlap of the incisors, distance from upper incisor tip to lower incisor tip	2.6 ± 3.5
20	Overjet (mm)	Horizontal overlap of the incisors, distance from upper incisor tip to labial surface of lower incisor	3.6 ± 3.4
21	Occlusal plane angle, OP–XiPm (^0^)	Angle between occlusal and Xi–Pm planes	22 ± 4.8
Soft tissue measurements			
22	Nasolabial angle (^0^)	The angle between two lines passing through the lower edge of the nose and the edge of the upper lip	94.7 ± 9.9
23	Chin position, Pog’–TVL (mm)	Distance of Pog’ to TVL	−4.5 ± 1.5

SD, standard deviation; CC, cranial centris point; N, nasion; Cp, condylion-posterior point; PtV, vertical articulare pterygoid line; Ba, basion; FH, Frankfort horizontal plane; A, A-point; S, sella; Co, condylion-superior point; B, B-point; Xi, Xi point; Pm, protuberance menti; Pt, pterygoid; Gn, gnathion; Pog, pogonion; ANS, anterior nasal spine; PNS, posterior nasal spine; IMPA, incisor mandibular plane angle; TVL, true vertical line; Pog’, pogonion soft tissue.

#### b) Protocols of orthognathic surgery.

The orthognathic surgery protocol is summarized in S3 Fig. In brief, it consisted of three main phases: pre-surgical orthodontic treatment to align the dentition and decompensate occlusion; surgical intervention including bilateral sagittal split osteotomy (BSSO) for mandibular correction and Le Fort I osteotomy for maxillary repositioning as indicated; and post-surgical orthodontic treatment to refine occlusion, finalize interdigitation, and ensure long-term stability.

### Cephalometry and cast model analysis

Dentoskeletal and soft tissue changes were evaluated using cephalometric analysis. Frontal and lateral cephalograms were obtained for all patients at two time points: before treatment initiation and after treatment completion. To ensure objectivity, cephalograms were anonymized and randomly assigned identification numbers prior to analysis. All tracings and measurements were conducted using Webceph software (Webceph; https://webceph.com, Assemblecircle Corp., Korea).

**Fig 1:**
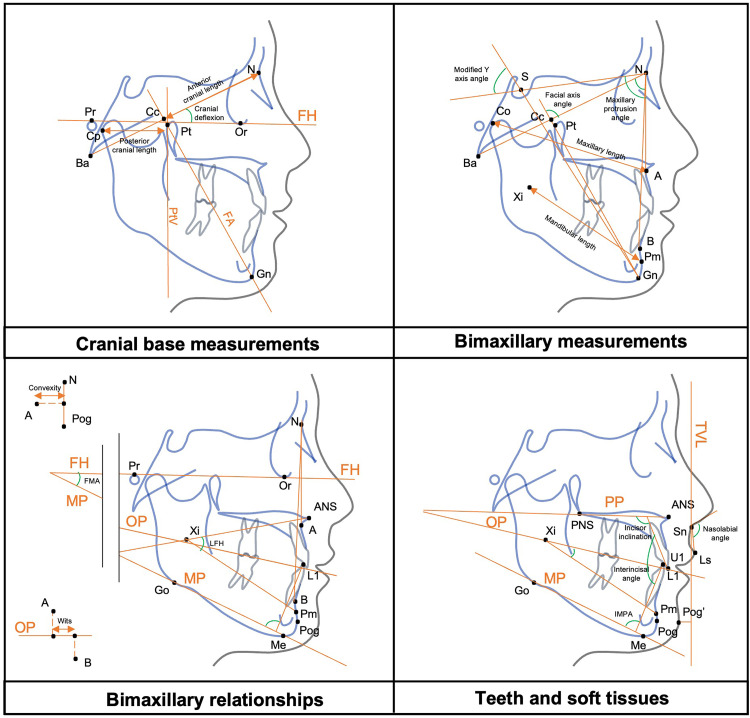
Dentoskeletal and soft tissue measurements on cephalometric analysis. CC, Center of cranium point; Ba, basion; N, nasion; Cp, condylion-posterior point; Pt, pterygoid; PtV, vertical articulare pterygoid line; Pr, Porion; Or, Orbitale; FH, Frankfort horizontal plane; Gn, gnathion; FA, facial axis; S, sella; A, A-point; Co, condylion-superior point; B, B-point; Xi, Xi point; Pm, protuberance menti; Pog, pogonion; Me, menton; OP, occlusal plane; MP, mandibular plane; FMA, Frankfort-mandibular plan angle; LFH, lower facial height; Wits, Wits appraisal; ANS, anterior nasal spine; PNS, posterior nasal spine; PP, palatal plane; U1, upper incisor tip; L1, lower incisor tip; IMPA, incisor mandibular plane angle; TVL, true vertical line; Pog’, pogonion soft tissue; Ls, labrale superius; Sn, subnasale.

The key dentoskeletal and soft tissue parameters assessed in this study, along with Vietnamese normative references, are summarized in Table 1 [18,19]. Ricketts analysis was employed to evaluate parameters related to the cranial base, maxilla, mandible, dentition, and occlusal plane (Fig 1). Soft tissue evaluations were conducted using Arnett analysis on lateral cephalograms, while Sato’s method was utilized to analyze facial asymmetry and deviation through frontal cephalograms [7].

The severity of malocclusion was assessed pre-treatment using the Discrepancy Index (DI) in accordance with the American Board of Orthodontics (ABO) guidelines [20]. A DI score of 7–15 indicated mild malocclusion, 16–24 moderate malocclusion, and ≥25 severe malocclusion. Case completion was evaluated post-treatment using the Objective Grading System (OGS), with scores ≤27 considered acceptable according to ABO standards [21].

To quantify measurement error, the Dahlberg formula was applied [22]. Ten cephalometric radiographs and ten mounted models were randomly selected and remeasured by the same investigator after a two-week interval. For cephalometric analysis, the FMA angle (°) was chosen as a representative angular measurement, as it involves multiple landmarks and is prone to tracing variability. For dental model analysis, overjet (mm) was selected as a representative linear measurement. The Dahlberg error was 0.91° for FMA and 0.93 mm for overjet, indicating high measurement reliability, as values <1° or <1 mm are considered clinically acceptable [23]. Raw cephalometric measurements used for analysis are provided in S2 File.

### Facial aesthetics evaluation

Facial profile and smile esthetics were evaluated using standardized photographs taken before and after treatment. Images were captured under controlled conditions with a fixed camera distance of 150 cm, consistent lighting, and a neutral gray background. Patients maintained a natural head position with the Frankfort horizontal (FH) plane parallel to the floor, eyes open, looking forward, and ears exposed for orientation. Patients were instructed to produce a natural smile. The camera was positioned at eye level and perpendicular to the facial midline. Each photograph included the full head and neck, properly aligned for frontal and profile views.

Image quality followed the American Board of Orthodontics (ABO) standards [24]. For frontal views, the interpupillary line was kept horizontal and the nose tip centered; for profile views, teeth were in occlusion, lips relaxed, and the left eyelash slightly visible. All photographs were reviewed to ensure sharp focus, balanced exposure, even illumination, and natural color tone before recording.

Adobe Photoshop CSE Extended® version 2020 was applied to minimize variation in hairstyle, adjust brightness and contrast, and unify the background to white. Shadowing and black-and-white conversion were applied to minimize potential bias from skin tone or other visual distractions. To ensure anonymization, the patients’ eyes were obscured.

Aesthetic assessments were conducted by two independent panels of three raters each: orthodontists (>5 years’ experience, mean age 32.3 years; 2 females, 1 male) and laypersons (non-medical professionals, mean age 35.3 years; 2 males, 1 female) recruited by convenience sampling [25]. Each patient contributed four photographs: frontal smiling and profile views at pre- and post-treatment. The photographs were anonymized, coded, and presented in random order to ensure raters were blinded to both treatment allocation and treatment time point.

A 0–10 Likert scale [26] was used, where 0 indicated ‘Extremely unattractive’ and 10 indicated ‘Very attractive’ (S4 Fig). Evaluations were conducted over five sessions without formal calibration; however, all raters received standardized written instructions. Raters were instructed to focus on overall facial balance, smile, and profile, and to disregard individual features such as the nose or hairstyle. To assess intra-rater reliability, 10 randomly selected photographs were re-evaluated across the five sessions. Likert scores were converted into three nominal categories (0–3 = unattractive, 4–7 = acceptable, 8–10 = harmonious) for analysis. Weighted Kappa was used with a minimum acceptable threshold of 0.6 [27]. All raters exceeded this threshold, with an overall weighted Kappa of 0.68, indicating substantial agreement. Inter-rater reliability was further assessed using Fleiss’ Kappa based on 10 randomly selected facial profile images (5 male, 5 female). Agreement among orthodontists was moderate (κ = 0.52, 95% CI: 0.17–0.88, *p* < 0.01), while the layperson panel showed slight agreement (κ = 0.17, 95% CI: –0.14 to 0.48, *p* > 0.05).

### Statistical analysis

Data analysis was conducted using SPSS (version 28, IBM, New York, USA). The Kolmogorov–Smirnov test was used to evaluate the normality of data distribution. Parametric tests were applied for normally distributed variables.

Quantitative variables were expressed as mean ± standard deviation, and categorical variables as proportions. Paired t-tests compared pre-treatment and post-treatment outcomes within groups, while independent t-tests compared variables between groups. One-sample t-tests assessed treatment outcomes against Vietnamese normative values. Pearson correlation evaluated associations between dentoskeletal variables. ANCOVA compared post-treatment outcomes between groups, adjusting for baseline mandibular length, sex, and tooth extraction. Multiple linear regression identified factors associated with changes in the anteroposterior skeletal relationship. A *p*-value < 0.05 was considered statistically significant.

## Results

### Clinical complexity and treatment outcome

**Fig 2:**
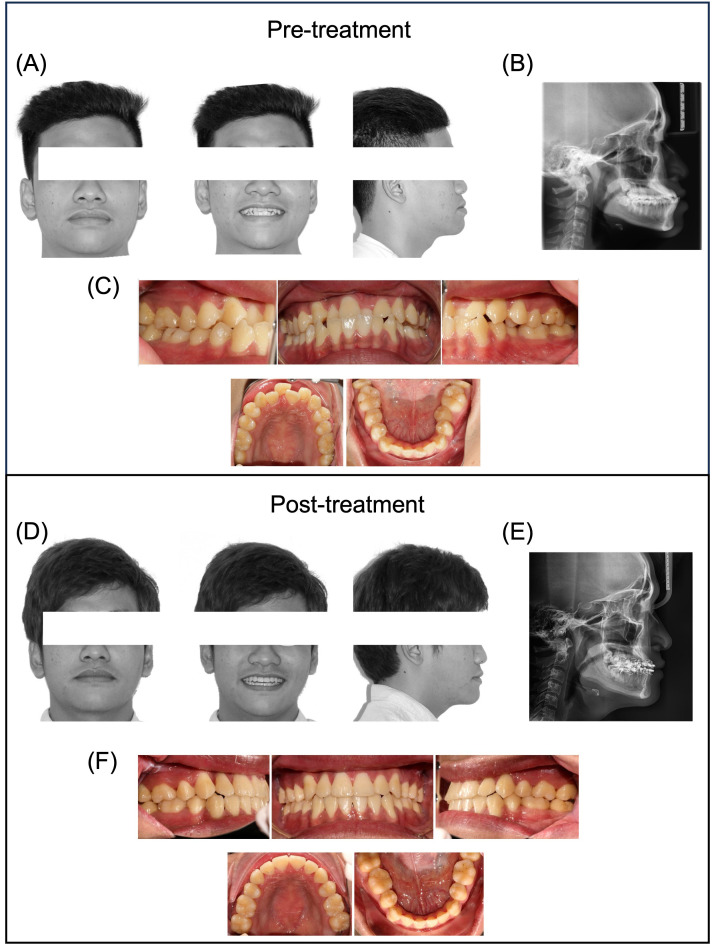
Representative patient treated with the MEAW technique. (A–C) Pre-treatment: (A) Frontal (with and without smiling) and profile view; (B) Lateral cephalogram; (C) Intraoral view. (D–F) Post-treatment: (D) Frontal (with and without smiling) and profile view; (E) Lateral cephalogram; (F) Intraoral view.

The clinical complexity of 30 skeletal Class III malocclusion cases treated with MEAW was assessed using the ABO-DI, yielding an average DI score of 35.8 ± 9.9. Of these, 13.3% (4 cases) were classified as moderate, and 86.7% (26 cases) were categorized as severe. There was no significant difference in the severity of malocclusion between male and female patients, as assessed by the DI score (*p* > 0.05). Post-treatment, a high case completion rate of 97% (29/30 cases) was achieved, with an average OGS score of 18.1 ± 5.9. The mean overjet was also noted to increase significantly by 4.3 mm, achieving an average of 3.1 mm post-treatment (*p* < 0.001; Table 2). Functional occlusion was successfully attained after treatment (Fig 2).

**Table 2:** Dentoskeletal changes before and after treatment in the MEAW group (n = 30).

Variables	Pre-treatment (mean ± SD)	Post-treatment (mean ± SD)	95% CI of the difference	*p*-value
Cranial base measurements				
CcN	56.4 ± 1.9	56.8 ± 2	−0.25–0.98	0.237
Cp–PtV	32.4 ± 2.5	33.1 ± 2.8	0.05–1.31	0.035*†
BaN–FH	27.5 ± 1.8	27.7 ± 1.7	−0.46–0.74	0.636
Maxillary measurements				
BaNA	61.7 ± 3.4	62.7 ± 3.3	0.51–1.49	<0.001***
SNA	82.2 ± 3.3	82.8 ± 3.2	−0.06–1.25	0.074
CoA	85.5 ± 3.9	87 ± 3.3	0.41–2.52	0.008**
Mandibular measurements				
SNB	84.7 ± 3.2	83.6 ± 3.5	−1.76 – −0.30	0.007**
XiPm	80.9 ± 5.6	80.8 ± 4.1	−1.60–1.42	0.908
PtGn–BaN	90.7 ± 3.6	90.3 ± 3.9	−0.83–0.10	0.12
SN–SGn	65.3 ± 3.3	66.1 ± 3.4	0.28–1.32	0.004**†
Anteroposterior relationships				
A–NPog	−3 ± 2.2	−1.6 ± 2.5	0.84–1.97	<0.001***
Wits	−8.9 ± 4.3	−5.2 ± 3	2.44–4.91	<0.001***
ANB	−2.5 ± 1.7	−0.9 ± 2	1.12–2.13	<0.001***
Vertical relationships				
LFH	46.4 ± 4.6	47 ± 4.6	−0.16–1.42	0.113
FMA	26.3 ± 6	26.4 ± 5.5	−0.88–1.12	0.811
Teeth and occlusal plane				
U1–PP	122.5 ± 7.8	124.2 ± 6.6	−1.37–4.70	0.27
IMPA	83.6 ± 7.5	80.5 ± 8.5	−6.04 – −0.21	0.036*
U1–L1	129.1 ± 13	130.1 ± 8.5	−3.42–5.43	0.646
Overbite	0.7 ± 2.7	1.2 ± 0.8	−0.40–1.53	0.238
Overjet	−1.2 ± 1.9	3.1 ± 1	3.59–5.06	<0.001***
OP–XiPm	25 ± 4.4	26.2 ± 4.1	−0.28–2.82	0.106
Soft tissue measurements				
Nasio-labial A	79.1 ± 8.6	79.8 ± 6.8	−2.44–3.72	0.675
Pog’–TVL	2.5 ± 4.8	1.8 ± 4.5	−1.48–0.01	0.052

SD, standard deviation; CI, confidence interval; Cc, cranial centris point; N, nasion; Cp, condylion-posterior point; PtV, vertical articulare pterygoid line; Ba, basion; FH, Frankfort horizontal plane; A, A-point; S, sella; Co, condylion-superior point; B, B-point; Xi, Xi point; Pm, protuberance menti; Pt, pterygoid; Gn, gnathion; Pog, pogonion; LFH, lower facial height; FMA, Frankfort-mandibular plane angle; PP, palatal plane; U1, upper incisor tip; L1, lower incisor tip; IMPA, incisor mandibular plane angle; OP, occlusal plane; TVL, true vertical line; Pog’, pogonion soft tissue.

*p-value* *< 0.05; **p-value* *< 0.01; ***p-value* *< 0.001 assessed by paired-samples t test.

† Statistically significant but with a change <1 mm or <1°.

**Fig 3:**
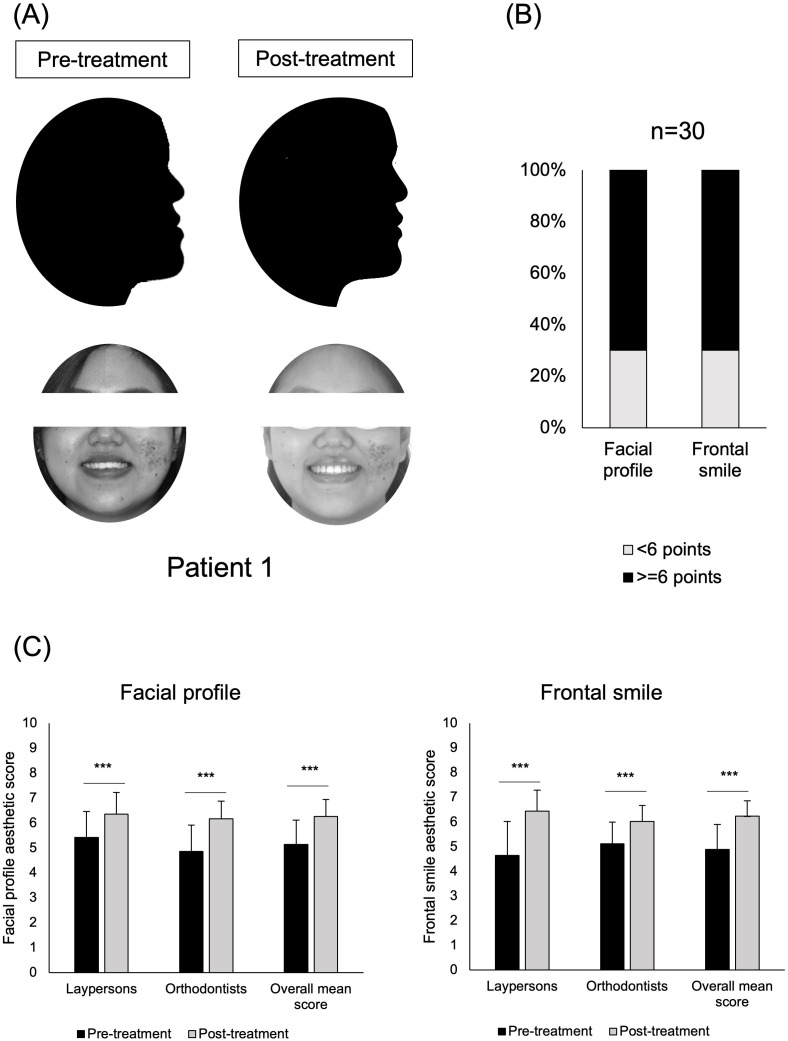
Aesthetic changes before and after treatment. (A) Profile and frontal smiling views of a representative patient with skeletal class III malocclusion before and after treatment. (B) Profile and frontal smile aesthetic scores after treatment were evaluated using Likert scale (0-10). (C) Evaluation of aesthetic changes before and after treatment by laypersons and orthodontists. Data are presented as a bar chart. *p value <0.05; **p value <0.01 assessed by paired-sample t-test.

Aesthetic outcomes were also significantly improved, with average scores for facial profile and frontal smile after treatment being 6.3 and 6.2, respectively. Facial aesthetics showed significant improvement after treatment, transitioning from “unattractive” to “acceptable-attractive” level (*p* < 0.001; Fig 3A). The result showed that 97% of patients achieved an “acceptable” level (≥6 score) in either profile or smile aesthetics, with 13 out of 30 patients (43.33%) attaining the level in both (Fig 3B). This improvement was consistently observed by both laypersons and orthodontists (*p* < 0.001; Fig 3C).

In the surgical group (n = 30), facial profile and frontal smile scores also showed significant post-treatment improvement as expected (*p* < 0.001). Post-treatment facial profile scores did not differ significantly between MEAW and surgical groups (*p* > 0.05; S5 Fig). Notably, the MEAW group achieved higher frontal smile scores as rated by laypersons compared with the surgical group (*p* < 0.01, S5 Fig).

Regarding differences among raters, interestingly, orthodontists tended to assign lower facial profile scores and higher frontal smile scores than laypersons before treatment; however, this discrepancy was no longer observed after treatment (*p* > 0.05, S5 Fig).

### Dentoskeletal and soft tissue changes

Cephalometric analyses were conducted to investigate the dentoskeletal changes in patients with skeletal Class III malocclusion treated using the MEAW technique. Post-treatment, significant alterations were observed in several dentoskeletal parameters (Table 2).

In the dental parameters, overjet improved significantly while overbite remained unchanged (*p* > 0.05). Upper incisor proclination showed no significant change (*p* > 0.05), whereas lower incisors exhibited retroclination. However, no significant differences were observed in the interincisal angle between pre- and post-treatment measurements (*p* > 0.05). The occlusal plane angle, evaluated relative to the Xi-Pm planes, also showed no significant change (*p* > 0.05).

Skeletal changes were noted primarily in the cranial base and bimaxillary regions. Adjustments in the cranial base were limited to posterior shifts (*p* < 0.05), with no anterior changes detected (*p* > 0.05). Maxillary measurements revealed significant increases in the BaNA angle and effective maxillary length (*p* < 0.01), although changes in the SNA angle were not significant (*p* > 0.05). Mandibular modifications included decreases in the SNB angle and increases in the modified Y-axis angle (*p* < 0.01), while the facial axis angle and mandibular length remained stable (*p* > 0.05).

In the soft tissue evaluation, chin retrusion, assessed by Pog’-TVL, decreased by an average of 0.7 mm, but this change was not statistically significant (*p* = 0.052; Table 2). No significant alterations were observed in the nasolabial angle (*p* > 0.05).

Post-treatment outcomes, adjusted for covariates including sex, extraction status, and mandibular length, are summarized in Table 3. Most dentoskeletal parameters did not differ significantly between the MEAW and surgical treatment groups. However, the surgical group demonstrated greater improvement in the ANB angle, with additional significant differences observed in posterior cranial length, maxillary protrusion angle, SNB angle, and interincisal angle. Enhanced improvement in anterior bimaxillary relationships in the surgical group compared with the MEAW group was clearly shown in the detailed dentoskeletal changes (*p* < 0.001; S2 Table).

**Table 3:** Comparison of post-treatment outcomes between MEAW and surgical groups.

Variables	MEAW group		Surgical group		*p*-value
	Unadjusted mean (SD)	Adjusted mean (95% CI)	Unadjusted mean (SD)	Adjusted mean (95% CI)	
Cranial base measurements					
CC-Na	56.8 (2)	56.5 (55.52 – 57.47)	53.7 (3.3)	54.0 (53.07–55.02)	0.002**
Cp–PtV	33.1 (2.8)	32.9 (31.68–34.05)	31 (3.1)	31.2 (30.02–32.39)	0.081
BaN–FH	27.7 (1.7)	27.8 (27.98 – 28.71)	28.7 (2.4)	28.5 (27.67–29.40)	0.318
Maxillary measurements					
BaNA	62.7 (3.3)	62.3 (60.93–63.70)	64.2 (3.3)	64.6 (63.21–65.98)	0.041*
SNA	82.8 (3.2)	82.7 (81.72–83.74)	83.9 (0.8)	83.9 (82.93–84.95)	0.133
CoA	87 (3.3)	86.5 (85.01–88.08)	87.3 (5.5)	87.7 (86.19–89.26)	0.334
Mandibular measurements					
SNB	83.6 (3.5)	83.6 (82.55–84.74)	81.2 (0.9)	81.2 (80.14–82.34)	0.007**
PtGn–BaN	90.3 (3.9)	89.6 (87.88–91.28)	88.6 (4.2)	89.3 (87.58–90.98)	0.825
SN–SGn	66.1 (3.4)	66.5 (64.95–67.97)	67 (3.8)	66.7 (65.16–68.18)	0.861
Anteroposterior relationships					
A–NPog	−1.6 (2.5)	−1.1 (−2.26–0.13)	0.8 (3.2)	0.2 (−0.95–1.43)	0.172
Wits	−5.2 (3)	−4.8 (−6.11 – −3.49)	−4.4 (3.2)	−4.8 (−6.09 – −3.47)	0.982
ANB	−0.9 (2)	−0.6 (−1.49–0.26)	1.1 (2.1)	0.9 (−0.01–1.74)	0.037*
Vertical relationships					
LFH	47 (4.6)	47.0 (45.18–48.74)	49.1 (3.8)	49.1 (47.35–50.92)	0.127
FMA	26.4 (5.5)	26.4 (24.30–28.56)	26 (4.3)	24.0 (23.83–28.10)	0.784
Teeth and occlusal plane					
U1–PP	124.2 (6.6)	123.8 (120.90–126.71)	124.6 (7.7)	125.0 (122.05–127.86)	0.618
IMPA	80.5 (8.5)	82.2 (78.77–85.72)	87.2 (8.4)	85.5 (82.03–88.98)	0.238
U1–L1	130.1 (8.5)	129.5 (125.97–132.98)	123 (9)	123.6 (120.12–127.12)	0.038*
Overbite	1.2 (0.8)	1.3 (0.88–1.72)	1.4 (1.1)	1.4 (0.96–1.80)	0.809
Overjet	3.1 (1)	3.0 (2.541–3.46)	3.1 (1.3)	3.2 (2.73–3.64)	0.608
OP–XiPm	26.2 (4.1)	25.5 (23.98–26.96)	24.9 (3.3)	25.6 (24.14–27.11)	0.894

SD, standard deviation; CI, confidence interval; CC, Center of cranium point; N, nasion; Cp, condylion-posterior point; PtV, vertical articulare pterygoid line; Ba, basion; FH, Frankfort horizontal plane; A, A-point; S, sella; Co, condylion-superior point; B, B-point; Pt, pterygoid; Gn, gnathion; Pog, pogonion; LFH, lower facial height; FMA, Frankfort-mandibular plane angle; PP, palatal plane; U1, upper incisor tip; L1, lower incisor tip; IMPA, incisor mandibular plane angle; OP, occlusal plane; Xi, Xi point; Pm, protuberance menti.

Adjusted means were estimated using ANCOVA, controlling for sex, extraction status, and mandibular length. *p-value* *< 0.05; **p-value* *< 0.01 indicate between-group comparisons after adjustment.

### Bimaxillary relationship improvements

**Fig 4:**
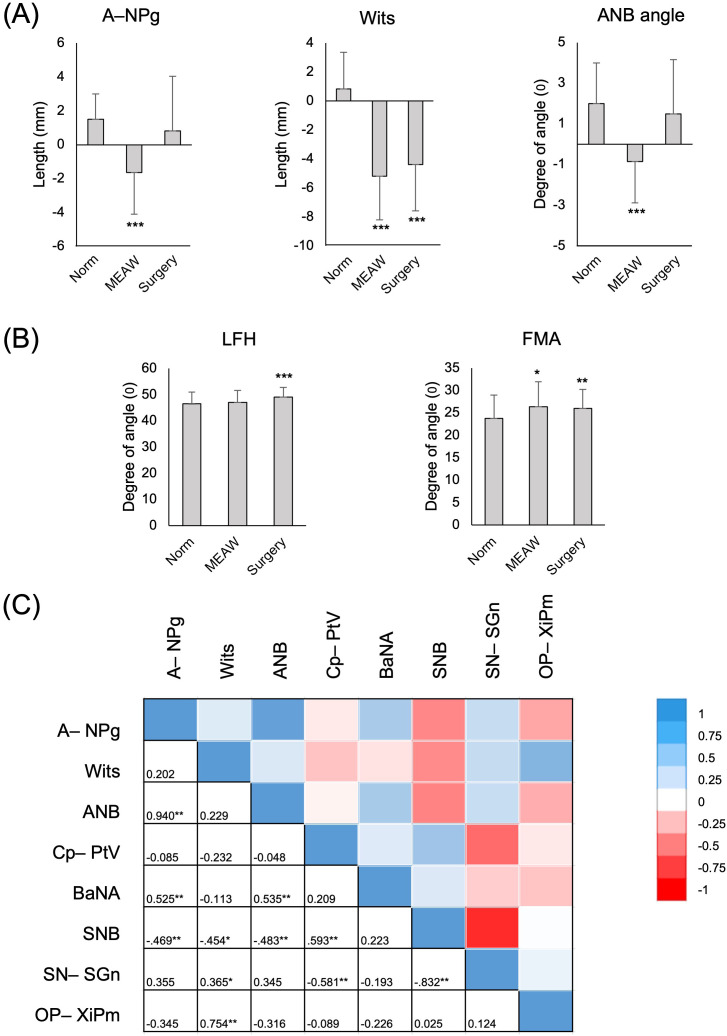
Changes in bimaxillary relationships after MEAW treatment and contributing impact factors. (A) Outcome of anteroposterior relationship changes in MEAW treatment and surgical comparison groups in comparison with normative values. (B) Outcome of vertical relationship changes in MEAW treatment and surgical comparison groups in comparison with normative values. Data are presented as a bar chart. *p value <0.05; **p value <0.01; ***p value <0.001 assessed by one-sample t-test. (C) Pearson’s correlation between anteroposterior relationship changes and skeletal factors. Unit on color scale: correlation value R. *p value <0.05; **p value <0.01. A–NPog, convexity; Wits, Wits appraisal; LFH, lower facial height; FMA, Frankfort-mandibular plane angle; Norm, normative value; Cp–PtV, posterior cranial length; OP–XiPm, occlusal plane angle, SN–SGn, modified Y axis.

The bimaxillary relationship, including vertical and anteroposterior dimensions, which influence the patient’s post-treatment profile, was evaluated. Post-treatment, patients treated with the MEAW technique showed significant improvements in anteroposterior skeletal relationships, evidenced by increases in convexity, Wits appraisal, and ANB angle (*p* < 0.001). However, when comparing post-treatment anteroposterior measurements with the surgical group, significant differences were observed, with the surgical group showing greater improvement in the ANB angle (*p* < 0.05; Table 3). Compared to normative values, the surgical group achieved more substantial improvements in the anteroposterior dimension, bringing measurements closer to the norm (*p* > 0.05), whereas the MEAW treatment showed less pronounced improvements (*p* < 0.001; Fig 4A).

Despite these notable anteroposterior changes, no significant alterations in vertical relationships were observed post-treatment in the MEAW group (*p* > 0.05; Table 2). Interestingly, while lower facial height (LFH) in the MEAW group did not differ significantly from normative values (*p* > 0.05), the surgical group exhibited higher LFH values compared to the norm (*p* < 0.01; Fig 4B).

### Factors influencing anteroposterior bimaxillary relationships

The improvement in the anteroposterior relationship in skeletal Class III malocclusion results from changes in both maxillary and mandibular factors. This study investigated correlations between changes in anteroposterior relationship parameters measured by convexity, Wits appraisal, and ANB angle, and various skeletal parameters to identify contributing factors. Significant correlations were observed between changes in anteroposterior relationship parameters and skeletal factors such as the SNB angle and BaNA angle (*p* < 0.05; Fig 4C). Additionally, the occlusal plane angle relative to the Xi-Pm planes and the modified Y-axis angle showed positive correlations with changes in Wits appraisal (*p* < 0.05). While no direct correlation was found between posterior cranial length changes and anteroposterior improvements, posterior cranial length was strongly associated with the modified Y-axis angle and SNB angle (*p* < 0.01; Fig 4C).

To further evaluate these contributions, a multiple linear regression model was applied, incorporating confounding factors such as gender, extraction status, complexity ABO-DI score, and incisor angle changes (Table 4). The analysis revealed that changes in posterior cranial length were associated with changes in convexity (β = 0.353; *p* < 0.05), Wits appraisal (β = 0.226; *p* < 0.05), and ANB angle (β = 0.436; *p* < 0.01). Similarly, changes in the BaNA angle significantly contributed to changes in convexity (β = 0.381; *p* < 0.05) and the ANB angle (β = 0.422; *p* < 0.01). Changes in incisor inclination angle were also associated with improvements in the anteroposterior relationship (*p* < 0.05). However, changes in the SNB angle, modified Y-axis angle, and occlusal plane angle were significantly correlated only with changes in Wits appraisal (*p* < 0.05).

**Table 4:** Multiple linear regression analysis for the change in the anteroposterior relationships in the patient with skeletal class III malocclusion treated with MEAW technique (n = 30).

Factors	B	Beta (β)	95% CI of B	*p*–value
A–NPog (R^2 ^= 0.794)				
Gender	0.475	0.108	−0.699–1.649	0.408
ABO-DI score	0.013	0.082	−0.026–0.051	0.501
Premolars extraction	−0.716	−0.12	−2.526–1.094	0.418
Cp–PtV	0.32	0.353	−0.025–0.614	0.035*
BaNA	0.444	0.381	0.074–0.814	0.021*
SNB	−0.257	−0.329	−0.704–0.19	0.244
SN–SGn	0.164	0.15	−0.369–0.698	0.527
OP–PmXi	−0.016	−0.045	−0.148–0.115	0.796
U1–PP	−0.074	−0.393	−0.139 – −0.008	0.03*
IMPA	0.086	0.444	−0.003–0.175	0.056
Wits (R^2^ = 0.925)				
Gender	0.383	0.04	−1.154–1.921	0.608
ABO-DI score	0.039	0.117	−0.011–0.089	0.121
Premolars extraction	1.131	0.087	−1.241–3.502	0.331
Cp–PtV	0.445	0.226	0.059–0.831	0.026*
BaNA	0.126	0.05	−0.36–0.611	0.594
SNB	−1.118	−0.658	−1.704 – −0.533	<0.001***
SN–SGn	−0.792	−0.333	−1.491 – −0.093	0.028*
OP–PmXi	0.802	1.011	0.63–0.974	<0.001***
U1–PP	−0.083	−0.203	−0.169–0.004	0.059
IMPA	0.148	0.35	0.032–0.265	0.016*
ANB (R^2^ = 0.814)				
Gender	0.35	0.09	−0.64–1.339	0.469
ABO-DI score	0.013	0.094	−0.019–0.045	0.417
Premolars extraction	0.172	0.032	−1.354–1.698	0.816
Cp–PtV	0.35	0.436	0.102–0.598	0.008**
BaNA	0.435	0.422	0.123–0.747	0.009**
SNB	−0.307	−0.444	−0.684–0.069	0.104
SN–SGn	0.077	0.08	−0.372–0.527	0.723
OP–PmXi	0.028	0.086	−0.083–0.139	0.604
U1–PP	−0.065	−0.395	−0.121 – −0.01	0.023*
IMPA	0.08	0.466	0.005–0.155	0.037*

B, regression coefficient; Beta, standardized coefficient; CI, confidence interval; R^2^, coefficients of determination. *p < 0.05; **p < 0.01; ***p < 0.001.

## Discussion

Skeletal Class III malocclusion represents a severe skeletal discrepancy that significantly impacts aesthetics and quality of life, often necessitating surgical intervention for aesthetic correction. While surgery is highly effective, increasing concerns about surgical risks and recovery have led to a growing interest in non-surgical alternatives [5]. This trend is particularly notable in Southeast Asia, where the high prevalence of Class III malocclusion has driven the development of less invasive orthodontic techniques [2]. Among these, the MEAW technique has gained popularity as a non-surgical approach for managing Class III malocclusion [10]. Despite its clinical success, evidence regarding the dentoskeletal changes induced by MEAW remains inconclusive. To address this gap, our study investigated the dentoskeletal changes and their contribution to improving bimaxillary relationships in Vietnamese patients treated with the MEAW technique. The results showed that, in addition to dental changes such as lower incisor retroclination, skeletal changes occurred in the cranial base and bimaxillary regions. Although the MEAW group did not achieve the same extent of skeletal correction as the surgical group, significant improvement in bimaxillary relationships, occlusal function, and overall facial aesthetics was observed [14,28,29].

From an aesthetic perspective, both groups showed marked post-treatment improvements in facial profile and frontal smile appearance as rated by laypersons and orthodontists. Interestingly, orthodontists tended to assign lower facial profile scores and higher frontal smile scores than laypersons before treatment. This indicates that orthodontists assessed facial esthetics more critically, particularly in profile evaluation, and generally spent more time focusing on the oral region than patients or laypersons [30,31]. In contrast, laypersons focused more on overall facial attractiveness and likely rated smiles less favorably when other facial features appeared unbalanced [31]. Pre-treatment differences between raters were no longer evident after treatment, indicating convergence in aesthetic judgment once facial balance and harmony were restored. Despite the greater skeletal correction in the surgical group, post-treatment facial profile scores did not differ significantly between groups, suggesting that MEAW can yield comparable aesthetic outcomes. Interestingly, laypersons assigned higher frontal smile scores to the MEAW group, possibly due to the predominance of female participants in this group, as gender-related preferences can influence perceived attractiveness. This pattern aligns with previous reports showing that females are more likely to seek aesthetic, non-surgical treatment options due to concerns about surgical risks [32]. In contrast, orthodontists evaluated aesthetics based on standardized criteria of facial harmony and professional norms, and their assessments were unlikely affected by gender. This was particularly evident in the facial profile assessments, which were based on shadowed photographs that minimized gender recognition; consequently, no post-treatment difference was found between rater groups. Moreover, inter-rater reliability analysis showed that orthodontists demonstrated greater consistency, whereas laypersons exhibited higher variability, reflecting the subjective and culturally influenced nature of aesthetic perception.

The improvement in bimaxillary relationships was a key outcome of MEAW treatment, aligning with one of the primary objectives of this modality. Post-treatment results revealed negligible changes in vertical dimensions for both the MEAW and surgical groups [14]. Notably, vertical parameters in the MEAW group were closer to normative values compared to the surgical group. These unchanged parameters are clinically acceptable. In Vietnamese Class III patients, vertical discrepancies are typically minimal compared to normative values, so treatment focuses on maintaining rather than altering vertical dimensions. The stable overbite post-treatment reflects appropriate vertical control aligned with treatment goals.

When evaluating anteroposterior relationships, as measured by convexity, Wits appraisal, and the ANB angle, significant improvements were observed. Although these values did not reach the ideal levels typically achieved through surgical intervention, remarkable enhancements in overjet and facial harmony were evident. These findings regarding bimaxillary relationships further support the effectiveness of MEAW in addressing bimaxillary discrepancies, particularly in correcting anteroposterior discrepancies. Differences between our treatment results and those reported in other MEAW studies likely reflect variations in case selection, as previous authors focused on open bite cases rather than skeletal Class III malocclusion [33–35].

The change in anteroposterior relationships, particularly in camouflage treatment, has been linked to dentoalveolar compensation [36]. In our study, multiple regression analysis showed that compensation in both jaws, represented by upper incisor inclination (U1–PP) and lower incisor inclination (IMPA), contributed to the anteroposterior correction, consistent with previous reports [37]. However, only the change in IMPA (~3°) was statistically significant, while the change in U1–PP (~2°) was not. A previous study suggested that during camouflage treatment, the angulation between the tooth axis and alveolar bone remains stable, indicating that the alveolar bone remodels according to incisor inclination [38]. Collectively, these findings indicate that anteroposterior improvement in skeletal Class III cases was mainly driven by mandibular rather than maxillary compensation [39,40]. The unchanged SNA angle supports limited maxillary involvement, whereas the decreased SNB angle reflects point B retrusion as the major contributor to the overall correction.

Regarding point B retrusion, the observed change may not result solely from dentoalveolar compensation but could also involve mandibular repositioning, such as clockwise rotation or backward movement. The MEAW technique is theoretically designed to induce mandibular backward repositioning through occlusal plane rotation, achieved by distal tipping of molars and extrusion and retraction of mandibular incisors [28]. The unchanged occlusal plane angle relative to the Xi–PM line in our study aligns with this concept of mandibular repositioning via occlusal plane adjustment. However, mandibular repositioning during MEAW treatment remains controversial, and clear evidence supporting true mandibular movement is limited. In this study, the facial axis angle showed no significant change, consistent with previous reports indicating minimal mandibular rotation [12,34]. To further evaluate mandibular displacement, we used the modified Y-axis angle, a more reliable indicator of mandibular rotation. Interestingly, this angle showed a statistically significant but small increase (<1°), suggesting minor mandibular repositioning with limited clinical relevance. The unchanged lower facial height (LFH) and Frankfort–mandibular plane angle (FMA) further support the absence of notable rotation. Therefore, if any mandibular repositioning occurred, it was likely minimal, with the backward shift potentially involving condylar displacement rather than rotation alone. Consequently, this backward shift could secondarily influence parameters such as posterior cranial base length. Although these linear changes were small, their inclusion in the regression model suggests a possible indirect contribution of cranial and condylar movement to overall anteroposterior correction. Supporting this interpretation, the facial angle and modified Y-axis values showed no significant differences between the MEAW and surgical groups, suggesting a comparable pattern of mandibular movement.

Beyond mandibular contributions, an increase in the BaNA angle, as demonstrated by Pearson correlation analysis, suggests that maxillary modifications may also have contributed to improvements in anteroposterior relationships. The observed increase in the BaNA angle could be attributed to either a forward shift of point A or a backward movement of the basion. While the unchanged cranial deflection and the slight increase in maxillary length (CoA) suggest some forward movement of point A, the lack of changes in the SNA angle indicates that such advancement was likely minimal. Therefore, it is hypothesized that the increase in BaNA angle may reflect a combination of forward movement of point A and posterior displacement of the basion. Notably, the persistence of unfused spheno-occipital and parietomastoid sutures into adulthood makes the backward movement of the basion anatomically plausible [41]. This potential displacement, possibly involving the posterior displacement of the occipital-temporal complex, could in turn affect the positions of the basion and lead to changes in BaN planes. These cranial plane changes might help explain why the facial axis angle remained unchanged, even if minor mandibular repositioning occurred.

These complex interactions may be partially explained by joint remodeling and craniofacial bone dynamics [42]. Craniofacial bones dynamically interact with skeletal sutures, even into adulthood [41], forming the foundation of the craniofacial dynamics theory [7]. This theory suggests that forces generated during occlusal function are transmitted via the masticatory muscles to the temporal bone through the temporomandibular joint, influencing cranial base bones and inducing flexion and extension movements. Consequently, orthodontic and prosthodontic treatments involving bite adjustments can have secondary effects on facial bones. This has been increasingly validated through studies on muscle-bone crosstalk in the craniofacial region, particularly with advancements in molecular biology [42]. Over the past decade, the concept of muscle-bone crosstalk has gained prominence, emphasizing the essential morphofunctional relationship between masticatory muscles and craniofacial bones [42,43]. Forces generated during chewing can deform the skeleton, eliciting cellular responses such as bone resorption or deposition [44]. These processes reshape bones to adapt to new loading scenarios, underscoring the dynamic interplay between form and function in craniofacial structures.

This study has several limitations. First, its retrospective design may introduce selection bias and limit causal inference. Second, potential measurement errors could arise from landmark identification and cephalometric tracing, and all measurements were performed by a single investigator, preventing assessment of inter-observer reproducibility. The relatively small sample size may also reduce the power to detect subtle intergroup differences. Third, although analyses were adjusted for baseline differences in sex, extraction status, and mandibular length, residual confounding from these factors cannot be completely excluded. Fourth, the small number of raters in the aesthetic evaluation limits the robustness of inter-rater reliability and formal hypothesis testing, and larger rater samples are recommended for future studies. Finally, hypotheses regarding condylar displacement or cranial base remodeling remain speculative and require confirmation using CBCT, longitudinal imaging, or TMJ assessment. Despite these limitations, the findings provide valuable insight into the biological complexity and skeletal interactions involved in orthodontic treatment and support the effectiveness of the MEAW technique as a camouflage approach for managing skeletal Class III malocclusion.

## Conclusions

Taken together, these findings suggest that anteroposterior improvement in this skeletal Class III cohort was primarily achieved through mandibular dentoalveolar compensation, with minor contributions from both mandibular and maxillary skeletal adaptations. These changes suggest complex interactions between dentoskeletal factors during non-surgical orthodontic treatment. Although the improvements were less pronounced than those achieved through surgical intervention, the MEAW technique may serve as a valuable treatment option for selected Class III patients, particularly when moderate skeletal correction and substantial aesthetic enhancement are desired. Further prospective, controlled studies are recommended to confirm its efficacy and assess long-term outcomes.

## Supporting information

S1 File STROBE Checklist. (PDF)

S2 File Raw data measurements. (XLSX)

S1 Fig Summary of the MEAW protocol. Treatment procedures were adjusted according to the vertical skeletal pattern, classified as low- or high-angle based on the lower facial height (LFH) index. Red arrows indicate step-down or step-up activations, and blue arrows indicate tip-back or tip-forward activations.
(JPG)

S2 Fig Summary of the MEAW protocol (continued). Treatment procedures were adjusted according to the vertical skeletal pattern, classified as low- or high-angle based on the lower facial height (LFH) index. Red arrows indicate step-down or step-up activations, and blue arrows indicate tip-back or tip-forward activations.
(JPG)

S3 Fig Orthognathic surgery protocol. (JPG)

S4 Fig Aesthetic evaluation method. (A) Processed frontal smiling and facial profile images used for scoring. (B) Corresponding 0–10 Likert scale, where 0 = ‘Extremely unattractive’ and 10 = ‘Very attractive.’.
(JPG)

S5 Fig Esthetic evaluation results. (A) Facial profile and frontal smile scores before treatment in MEAW and surgical groups. (B) Facial profile and frontal smile scores after treatment in MEAW and surgical groups. (C) Comparison of post-treatment esthetic scores between MEAW and surgical groups. Data are presented as bar chart. *p value <0.05; **p value <0.01 assessed by paired-sample t-test and independent-samples t-test.
(JPG)

S1 Table Baseline comparison of age and dentoskeletal measurements (mean ± SD) between the MEAW and surgical groups. (DOCX)

S2 Table Comparison of mean changes (Δ) between MEAW and surgical groups. (DOCX)
