# Supplementary material for: Dentoskeletal changes and anteroposterior improvements in skeletal class III malocclusion treated with MEAW: A retrospective study
Source: PLoS One. 2026 Jan 2;21(1):e0340197. doi: 10.1371/journal.pone.0340197 (PMC12758781; doi:10.1371/journal.pone.0340197)
Supplement: S1 File — (PDF) [file pone.0340197.s001.pdf]

STROBE Statement—checklist of items that should be included in reports of **cohort studies**

	Item No.	Recommendation	Page No.	Relevant text from manuscript
**Title and abstract**	1	(*a*) Indicate the study's design with a commonly used term in the title or the abstract	1	The title includes "A retrospective study".
		(*b*) Provide in the abstract an informative and balanced summary of what was done and what was found	2	See "Abstract" section in the manuscript.
**Introduction**				
Background/rationale	2	Explain the scientific background and rationale for the investigation being reported	3-4	See "Introduction" section in the manuscript.
Objectives	3	State specific objectives, including any prespecified hypotheses	4	See last paragraph of the "Introduction" section in the manuscript.
**Methods**				
Study design	4	Present key elements of study design early in the paper	2	See "Abstract" — Methods, first sentence.
Setting	5	Describe the setting, locations, and relevant dates, including periods of recruitment, exposure, follow-up, and data collection	4	See "Materials and Methods" — Patient selection, paragraph 2 in the manuscript.
Participants	6	(*a*) Give the eligibility criteria, and the sources and methods of selection of participants. Describe methods of follow-up	4-5	See "Materials and Methods" — Patient selection, paragraph 2 in the manuscript.
		(*b*) For matched studies, give matching criteria and number of exposed and unexposed	4-5	See "Materials and Methods" — Patient selection, paragraph 2 in the manuscript.<br>"The MEAW group consisted of 30 patients"<br>"The surgical group consisted of 30 patients"
Variables	7	Clearly define all outcomes, exposures, predictors, potential confounders, and effect modifiers. Give diagnostic criteria, if applicable	7-10	See Materials and Methods and Table 1 for definitions of all outcomes, exposures, predictors, and potential confounders. Diagnostic criteria for skeletal Class III malocclusion are provided in the inclusion criteria: ANB $< 0°$.
Data sources/ measurement	8*	For each variable of interest, give sources of data and details of methods of assessment (measurement). Describe comparability of assessment methods if there is more than one group	7-10	See "Materials and Methods" section in the manuscript.

Continued on next page

Bias	9	Describe any efforts to address potential sources of bias	6	Mandibular length, sex, and tooth extraction status were treated as covariates in the adjusted intergroup comparisons.
Study size	10	Explain how the study size was arrived at	4	A sample size calculation was performed using G*Power 3.1. See "Materials and Methods" — Patient selection, first paragraph.
Quantitative variables	11	Explain how quantitative variables were handled in the analyses. If applicable, describe which groupings were chosen and why	7-10	See "Materials and Methods" — Cephalometry and cast model analysis and Facial esthetics evaluation.
Statistical methods	12	(*a*) Describe all statistical methods, including those used to control for confounding	10	See "Materials and Methods" — Statistical analysis.
		(*b*) Describe any methods used to examine subgroups and interactions	10	"Multiple linear regression identified factors associated with changes in the anteroposterior skeletal relationship."
		(*c*) Explain how missing data were addressed	N/A	No missing data.
		(*d*) If applicable, explain how loss to follow-up was addressed	N/A	This is a retrospective study with no prospective follow-up period.
		(*e*) Describe any sensitivity analyses	N/A	N/A
**Results**				
Participants	13*	(a) Report numbers of individuals at each stage of study—eg numbers potentially eligible, examined for eligibility, confirmed eligible, included in the study, completing follow-up, and analysed	4-5	See "Materials and Methods" — Patient selection.
		(b) Give reasons for non-participation at each stage	N/A	N/A
		(c) Consider use of a flow diagram	N/A	N/A
Descriptive data	14*	(a) Give characteristics of study participants (eg demographic, clinical, social) and information on exposures and potential confounders	5-6	See "Materials and Methods" — Patient selection, last two paragraphs.
		(b) Indicate number of participants with missing data for each variable of interest	N/A	No missing data.
		(c) Summarise follow-up time (eg, average and total amount)	6	"The average treatment duration for the MEAW group was approximately 28 months, while the surgical group generally had a shorter overall treatment period."

Continued on next page

Outcome data	15*	Report numbers of outcome events or summary measures over time	11	See “Results” — Clinical complexity and treatment outcome.
Main results	16	(*a*) Give unadjusted estimates and, if applicable, confounder-adjusted estimates and their precision (eg, 95% confidence interval). Make clear which confounders were adjusted for and why they were included	28-29	See Table 2 and Table 3.
		(*b*) Report category boundaries when continuous variables were categorized	11	See “Results” — Clinical complexity and treatment outcome.
		(*c*) If relevant, consider translating estimates of relative risk into absolute risk for a meaningful time period	N/A	N/A
Other analyses	17	Report other analyses done—eg analyses of subgroups and interactions, and sensitivity analyses	30	Table 4
**Discussion**				
Key results	18	Summarise key results with reference to study objectives	14-15	See “Discussion” first paragraph, where key findings are summarized in relation to study aims.
Limitations	19	Discuss limitations of the study, taking into account sources of potential bias or imprecision. Discuss both direction and magnitude of any potential bias	19	See “Discussion” paragraph on study limitations.
Interpretation	20	Give a cautious overall interpretation of results considering objectives, limitations, multiplicity of analyses, results from similar studies, and other relevant evidence	15-19	See “Discussion” section in the manuscript.
Generalisability	21	Discuss the generalisability (external validity) of the study results	19	“The findings provide valuable insight into the biological complexity and skeletal interactions involved in orthodontic treatment and support the effectiveness of the MEAW technique as a camouflage approach for managing skeletal Class III malocclusion.”
**Other information**				
Funding	22	Give the source of funding and the role of the funders for the present study and, if applicable, for the original study on which the present article is based	31	There has been no significant financial support for this study.

*Give information separately for exposed and unexposed groups.

Note: An Explanation and Elaboration article discusses each checklist item and gives methodological background and published examples of transparent reporting. The STROBE checklist is best used in conjunction with this article (freely available on the Web sites of PLoS Medicine at http://www.plosmedicine.org/, Annals of Internal Medicine at http://www.annals.org/, and Epidemiology at http://www.epidem.com/). Information on the STROBE Initiative is available at www.strobe-statement.org.
