# Supplementary figures and images for: Dentoskeletal changes and anteroposterior improvements in skeletal class III malocclusion treated with MEAW: A retrospective study
Source: PLoS One. 2026 Jan 2;21(1):e0340197. doi: 10.1371/journal.pone.0340197 (PMC12758781; doi:10.1371/journal.pone.0340197)

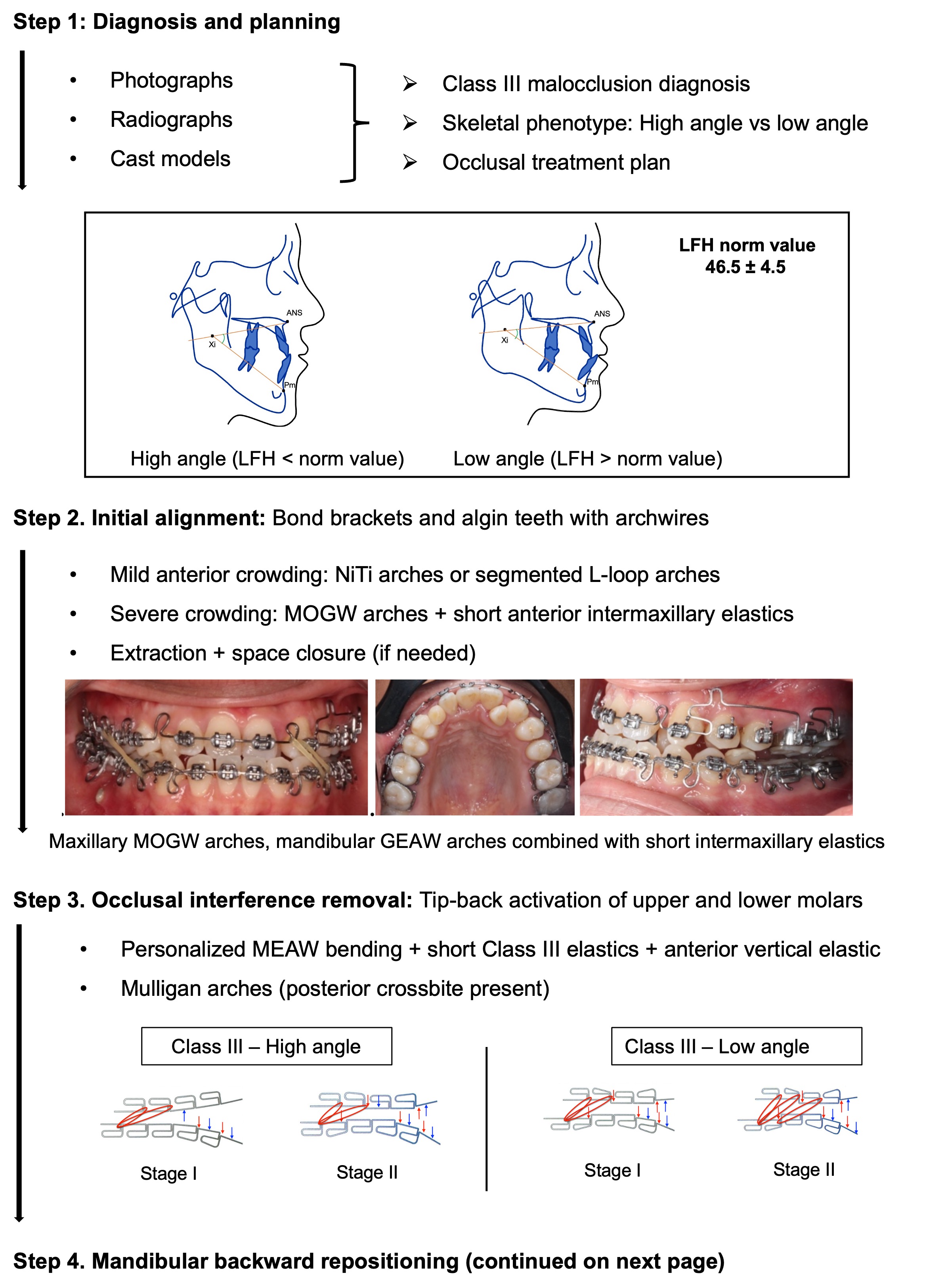

Supplement: S1 Fig — Treatment procedures were adjusted according to the vertical skeletal pattern, classified as low- or high-angle based on the lower facial height (LFH) index. Red arrows indicate step-down or step-up activations, and blue arrows indicate tip-back or tip-forward activations. (JPG) [file pone.0340197.s003.jpg]

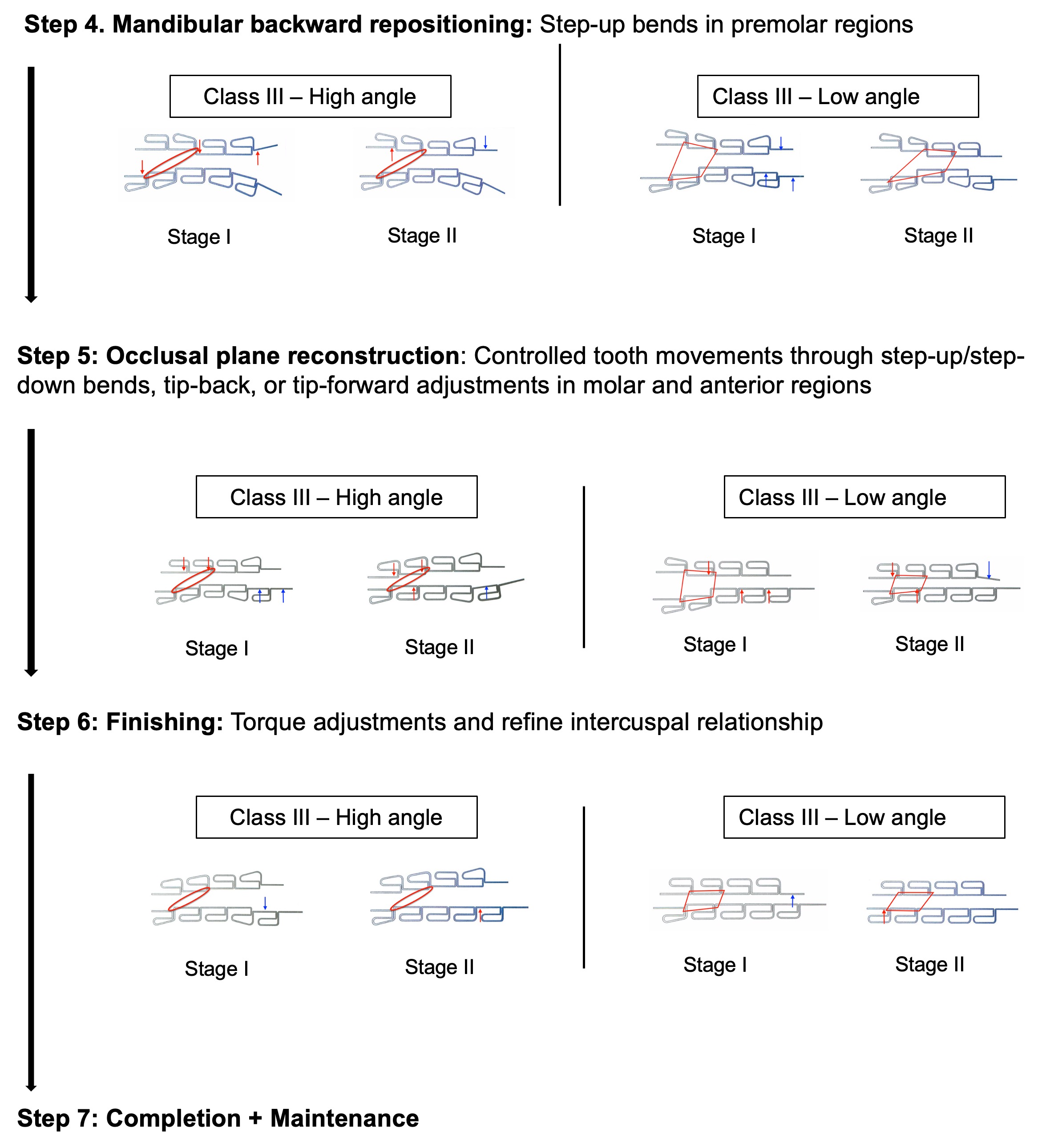

Supplement: S2 Fig — Treatment procedures were adjusted according to the vertical skeletal pattern, classified as low- or high-angle based on the lower facial height (LFH) index. Red arrows indicate step-down or step-up activations, and blue arrows indicate tip-back or tip-forward activations. (JPG) [file pone.0340197.s004.jpg]

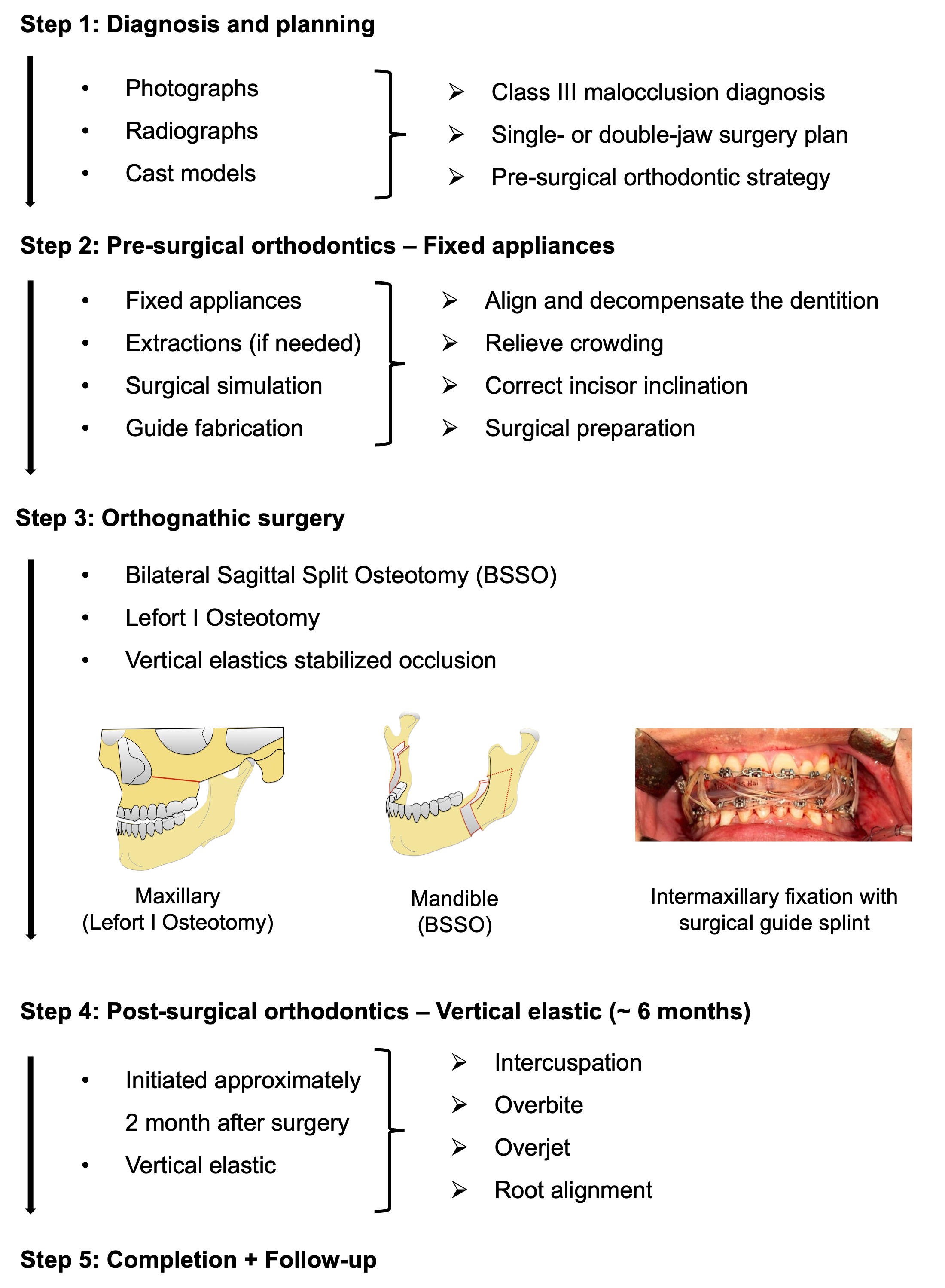

Supplement: S3 Fig — (JPG) [file pone.0340197.s005.jpg]

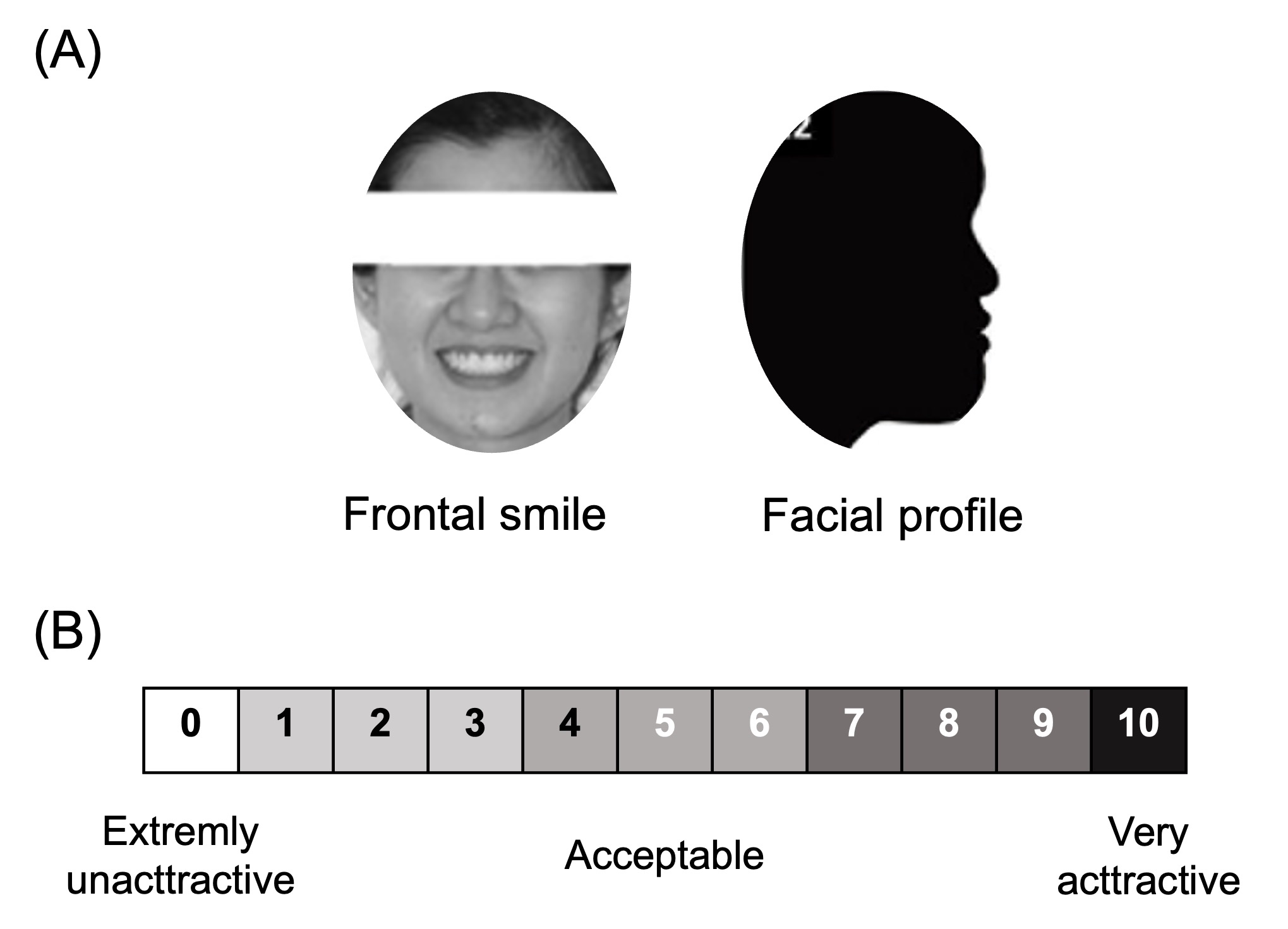

Supplement: S4 Fig — (A) Processed frontal smiling and facial profile images used for scoring. (B) Corresponding 0–10 Likert scale, where 0 = ‘Extremely unattractive’ and 10 = ‘Very attractive.’. (JPG) [file pone.0340197.s006.jpg]

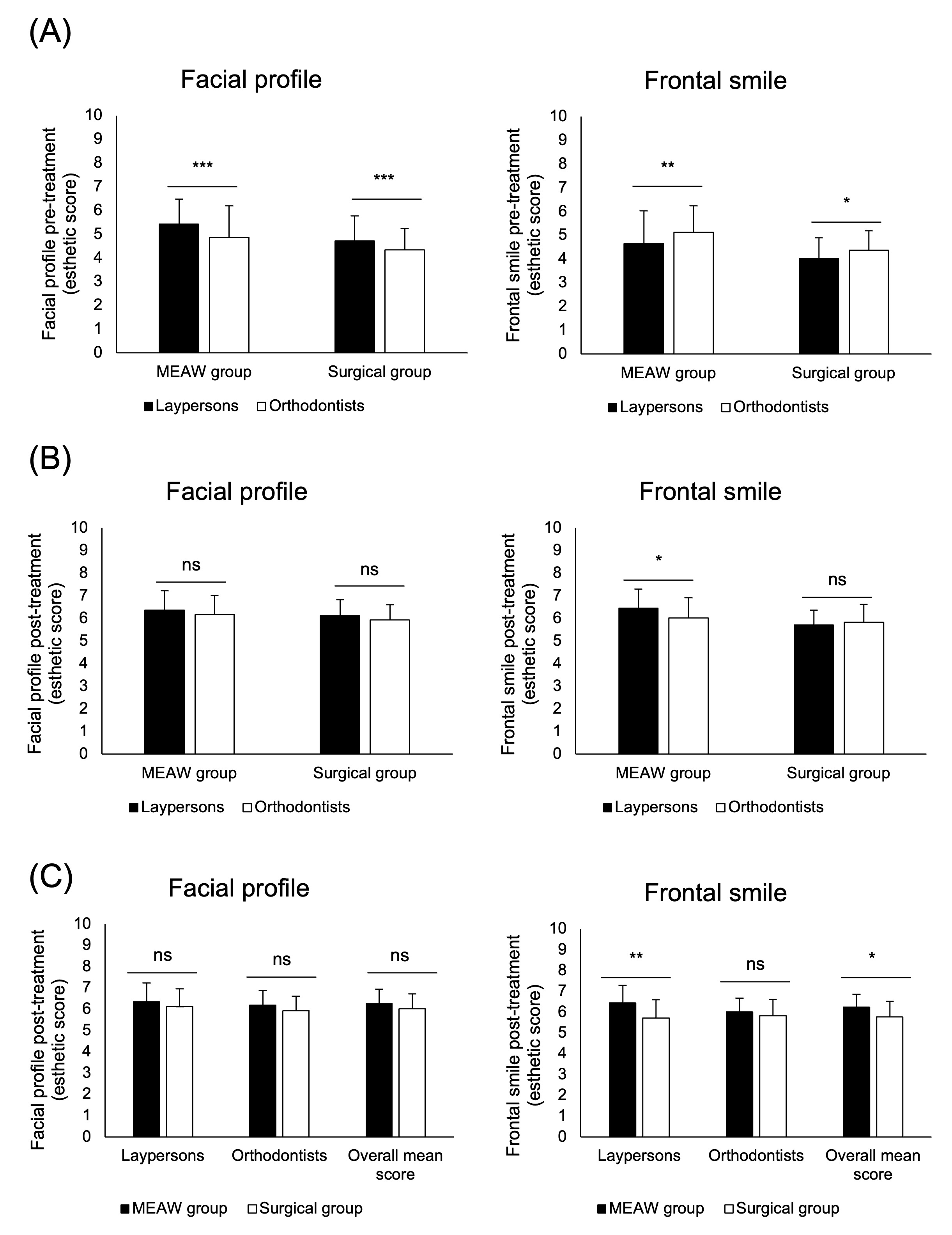

Supplement: S5 Fig — (A) Facial profile and frontal smile scores before treatment in MEAW and surgical groups. (B) Facial profile and frontal smile scores after treatment in MEAW and surgical groups. (C) Comparison of post-treatment esthetic scores between MEAW and surgical groups. Data are presented as bar chart. *p value <0.05; **p value <0.01 assessed by paired-sample t-test and independent-samples t-test. (JPG) [file pone.0340197.s007.jpg]
