# Supplementary material for: Dentoskeletal changes and anteroposterior improvements in skeletal class III malocclusion treated with MEAW: A retrospective study
Source: PLoS One. 2026 Jan 2;21(1):e0340197. doi: 10.1371/journal.pone.0340197 (PMC12758781; doi:10.1371/journal.pone.0340197)
Supplement: S1 Table — (DOCX) [file pone.0340197.s008.docx]

**S1 Table.** Baseline comparison of age and dentoskeletal measurements (mean ± SD) between the MEAW and surgical groups

| **Variables** | **MEAW group (n=30)** | **Surgical group (n=30)** | **95% CI of the difference** | ***p*-value** |
| --- | --- | --- | --- | --- |
| Age | 24.4 ± 6.4 | 22.7 ± 4.2 | -1.02 – 4.56 | 0.209 |
| Maxillary measurements | | | | |
| BaNA | 61.7 ± 3.4 | 62.4 ± 2.9 | -2.35 – 0.89 | 0.369 |
| CoA | 85.5 ± 3.9 | 85.7 ± 5 | -2.45 – 2.14 | 0.894 |
| Mandibular measurements | | | | |
| XiPm | 80.9 ± 5.6 | 76.2 ± 6.3 | 1.55 – 7.73 | 0.004** |
| PtGn–BaN | 90.7 ± 3.6 | 90.6 ± 4.4 | -2 – 2.16 | 0.938 |
| Anteroposterior relationships | | | | |
| A–NPog | -3 ± 2.2 | -3.3 ± 4 | -1.37 – 1.98 | 0.714 |
| ANB | -2.5 ± 1.7 | -3.5 ± 2.6 | -0.2 – 2.08 | 0.104 |
| Vertical relationships | | | | |
| LFH | 46.4 ± 4.6 | 48.3 ± 3.9 | -4.11 – 0.32 | 0.092 |
| FMA | 26.3 ± 6 | 26.1 ± 4 | -2.5 – 2.76 | 0.923 |
| Teeth | | | | |
| U1–PP | 122.5 ± 7.8 | 125.9 ± 5.7 | -6.94 – 0.13 | 0.059 |
| IMPA | 83.6 ± 7.5 | 81.9 ± 9 | -2.52 – 6.05 | 0.412 |
| U1–L1 | 129.1 ± 13 | 127.1 ± 10.2 | -4.08 – 8 | 0.518 |
| Overbite | 0.7 ± 2.7 | 0.6 ± 2 | -1.16 – 1.28 | 0.925 |
| Overjet | -1.2 ± 1.9 | -1.9 ± 2.8 | -0.62 – 1.85 | 0.323 |

SD, standard deviation; Ba, basion; N, nasion; Co, condylion-superior point; A, A-point; B, B-point; Xi, Xi point; Pm, protuberance menti; Pt, pterygoid; Gn, gnathion; Pog, pogonion; LFH, lower facial height; FMA, Frankfort-mandibular plane angle; PP, palatal plane; U1, upper incisor tip; L1, lower incisor tip; IMPA, incisor mandibular plane angle. ***p-*value < 0.01 assessed by an independent-samples t-test for comparison between the 2 groups at pre-treatment.
