# Supplementary material for: Dentoskeletal changes and anteroposterior improvements in skeletal class III malocclusion treated with MEAW: A retrospective study
Source: PLoS One. 2026 Jan 2;21(1):e0340197. doi: 10.1371/journal.pone.0340197 (PMC12758781; doi:10.1371/journal.pone.0340197)
Supplement: S2 Table — (DOCX) [file pone.0340197.s009.docx]

**S2 Table.** Comparison of mean changes (Δ) between MEAW and surgical groups

| **Variables** | **MEAW Δ change**  **(Mean ± SD)** | **Surgical Δ change**  **(Mean ± SD)** | ***p*-value**  **(Δ change)** |
| --- | --- | --- | --- |
| Cranial base measurements | | | |
| CcNa | 0.4 ± 1.6 | -0.1 ± 0.4 | 0.185 |
| Cp–PtV | 0.7 ± 1.7 | 0.4 ± 1.3 | 0.417 |
| BaN–FH | -0.1 ± 1.6 | -0.1 ± 0.7 | 0.487 |
| Maxillary measurements | | | |
| BaNA | 1 ± 1.4 | 1.8 ± 2.7 | 0.141 |
| SNA | 0.6 ± 1.8 | 1.3 ± 1.7 | 0.11 |
| CoA | 1.5 ± 2.8 | 1.6 ± 3.9 | 0.843 |
| Mandibular measurements | | | |
| SNB | -1.0 ± 1.9 | -5 ± 1.9 | <0.001*** |
| PtGn–BaN | -0.4 ± 1.3 | -2 ± 2.4 | 0.002** |
| SN–SGn | 0.8 ± 1.4 | 1.3 ± 2.5 | 0.341 |
| Anteroposterior relationships | | | |
| A–NPog | 1.4 ± 1.5 | 4.2 ± 2.9 | <0.001*** |
| Wits | 3.7 ± 3.3 | 9.2 ± 3.9 | <0.001*** |
| ANB | 1.7 ± 1.3 | 4.6 ± 2.7 | <0.001*** |
| Vertical relationships | | | |
| LFH | 0.6 ± 2.1 | 0.8 ± 2.4 | 0.823 |
| FMA | 0.1 ± 2.7 | -0.1 ± 3 | 0.724 |
| Teeth and occlusal plane | | | |
| U1–PP | 1.7 ± 8.1 | -1.3 ± 7.8 | 0.157 |
| IMPA | -3.1 ± 7.8 | 5.3 ± 6.6 | <0.001*** |
| U1–L1 | 1 ± 11.8 | -4.1 ± 9.5 | 0.071 |
| Overbite | 0.6 ± 2.6 | 0.8 ± 2.2 | 0.66 |
| Overjet | 4.3 ± 2 | 5 ± 2.9 | 0.318 |
| OP–XiPm | 1.3 ± 4.2 | 1.4 ± 3.1 | 0.861 |

SD, standard deviation; Δ, delta change; Cc, cranial centris point; N, nasion; Cp, condylion-posterior point; PtV, vertical articulare pterygoid line; Ba, basion; FH, Frankfort horizontal plane; A, A-point; S, sella; Co, condylion-superior point; B, B-point; Pt, pterygoid; Gn, gnathion; Pog, pogonion; LFH, lower facial height; FMA, Frankfort-mandibular plane angle; PP, palatal plane; U1, upper incisor tip; L1, lower incisor tip; IMPA, incisor mandibular plane angle; OP, occlusal plane; Xi, Xi point; Pm, protuberance menti. ***p-*value < 0.01; ****p-*value < 0.001 assessed by independent-samples t-test.
